# Cognitive functioning and prevalence of seizures among older persons in Uganda: A hospital-based, cross-sectional study

**DOI:** 10.1097/MD.0000000000031012

**Published:** 2022-10-14

**Authors:** Mark Kaddumukasa, Felix Bongomin, Levicatus Mugenyi, Micheal Kiyingi, Elly Katabira, Martha Sajatovic

**Affiliations:** a Department of Medicine, School of Medicine, College of Health Sciences, Makerere University, Kampala, Uganda; b Department of Immunology & Medical Microbiology, Faculty of Medicine, Gulu University, Gulu, Uganda; c Biostatistics Department, The AIDS Support Organisation (TASO), Kampala, Uganda; d Neurological and Behavioral Outcomes Center, University Hospitals Cleveland Medical Center & Case Western Reserve University School of Medicine, Cleveland, OH, USA.

**Keywords:** cognition, seizures, uganda

## Abstract

There is limited data on the prevalence of seizures and dementia among older persons in Uganda. We evaluated cognitive functioning, and the prevalence and factors associated with seizures among older persons attending an outpatient medical clinic in Uganda. We randomly selected older adults (60 years and above) attending Kiruddu National Referral Hospital medical outpatient clinics between October 2020 and March 2021. We excluded individuals with a history of head injury, brain tumors, mental retardation, co-morbidity with HIV and patients who have had recent brain surgery. Cognitive functioning was assessed using the Identification for Dementia in Elderly Africans (IDEA) tool. We enrolled 407 participants, with a median (inter-quartile range) age of 67 (64–73) years. Majority were female (n = 292, 71.7%). The prevalence of seizure was 1.5% (95% confidence interval [CI]: 0.7–3.3). All 6 participants reported generalized tonic-clonic seizure type. Self-reported seizure was associated with being female (adjusted prevalence ratio [aPR]: 0.79, 95%CI: 0. 67–0.93, *P* = .02) and residing in Mukono district (aPR: 17.26, 95%CI: 1.64–181.55, *P* = .018). Overall, 114 (28.1%) participants had cognitive deficit; 9 (2.2%) dementia and 105 (25.9%) impaired cognition. Cognitive deficit was independently associated with female gender (aPR: 0.61, 95%CI: 0.44–0.85, *P* = .003), formal employment (aPR: 0.53, 95%CI: 0.35–0.81, *P* = .003), age 70–74 (aPR: 1.69, 95%CI: 1.00–2.86, *P* = .049), and ≥ 75 years (aPR: 2.81, 95%CI: 1.71–4.61, *P* = .001). Prevalence of seizures among participants with cognitive deficit was 5.3% (6/114). Among older persons attending a medical clinic in Uganda, almost one-third had cognitive deficit with seizure prevalence being higher among these individuals.

## 1. Introduction

Neurological non-communicable diseases such as dementia and epilepsy are increasingly recognized in the developing world in tandem with the aging population.^[1]^ Epilepsy is the third most common neurological disorder affecting older adults after stroke and dementia.^[2]^ Considering the increasing aging population in sub-Saharan Africa, the overall incidence and prevalence of epilepsy is expected to increase with majority of the patients with newly diagnosed epilepsy being older adults.^[3–5]^ Furthermore, the characteristic symptoms of seizures in the elderly are different from those in younger patients.^[6,7]^

Epilepsy accounts for 0.75% of the global burden of disease and in 2012 epilepsy was attributed to approximately 20.6 million disability-adjusted life years lost.^[8,9]^ The estimated prevalence of epilepsy in Uganda is about 3% of the general population.^[10]^ However, no national surveys have been conducted, however, studies report an estimated prevalence ranging from 0.2% to 3.4% in different villages across the country.^[10]^ Epilepsy has significant economic implications in terms of health-care needs, premature death, and lost work productivity.^[11]^ The mortality of individuals with epilepsy is 2 to 3 times higher than that of the general population.^[12,13]^ People living with epilepsy have various diverse and complex effects on the overall well-being or subjective quality of life.^[14,15]^ Epilepsy is associated with profound physical, psychological and social consequences, and its impact on a person’s quality of life can be greater than that of some other chronic conditions.^[16]^

Alzheimer’s disease and related dementias (AD/ADRD), occur most frequently in the older adults.^[1]^ It is estimated that by 2030, the number of people aged 60 years and above will rise to over 67 million in sub-Saharan Africa.^[17]^ The prevalence of dementia within sub-Saharan Africa has been reported to range from 1.41% in an Ibadan community to 21.60% using the 10/66 diagnostic criteria compared to a prevalence rate of 6.40% when diagnosis was based on the DSM-IV criteria.^[18]^ The prevalence of dementia reported from 21 global burden of disease regions across the world ranges between 2.1% and 8.5% for those aged 60 years or older.^[19]^

Whereas the burden of AD/ARD in Uganda is not well established, few studies point to the existence of AD/ADRD with varying prevalence.^[20]^ AD/ADRD may also co-exist in the same individual with several other neurological non-communicable diseases conditions, 1 Ugandan study reported that 20% of stroke survivors had severe forms of dementia.^[21]^ Elderly individuals (aged at least 60 or older) represent a rapidly growing segment of the population in Uganda.^[22,23]^ However, data on epilepsy and AD/ADRD information is scanty in Uganda. This study aimed to determine cognitive functioning and the prevalence of seizure and associated factors among older persons in Uganda.

## 2. Methods

### 2.1. Study design & setting

This was a cross-sectional study conducted at the medical outpatient clinics of Kiruddu National Referral Hospital (KNRH), Kampala, Uganda between October 2020, and March 2021. KNRH draws a broad population, including substantial numbers of rural residents from across Uganda.

### 2.2. Study population

Participants were drawn from the medical outpatient clinics at KNRH. Eligible study participants were adults aged 60 years and more at the time of recruitment.

### 2.3. Inclusion and exclusion criteria

We included only persons of 60 years or older who provided written informed consent. Individuals with past medical history of head injury, brain tumors, mental retardation, co-morbid with HIV, and patients who had recent brain surgery were excluded from the study.

### 2.4. Study procedures

A standardized questionnaire was used to collect demographic, behavioral and medical history information, including ethnic group, marital status, and place of birth, residence, occupation, alcohol, and tobacco consumption.

### 2.5. Assessment of dementia

Brief dementia screening was conducted with the Identification for Dementia in Elderly Africans (IDEA) previously tested in SSA samples.^[24]^ The IDEA cognitive screen has been validated for use in populations with low levels of formal education within sub-Saharan Africa. The IDEA screen includes delayed recall, orientation, 2 measures of frontal lobe function, verbal fluency and abstract reasoning, praxis, and long-term memory. An assessment of ability for new learning is also possible from performance on the 10-word learning list. No items are included requiring reading, writing, drawing or calculation to reduce possible educational bias. The maximum possible score is 15 and the minimum 0, with a higher score indicating better cognitive function. Those who have low scores were objectively evaluated for dementia using Addenbrooke’s Cognitive Examination III performed. An Addenbrooke’s Cognitive Examination III score <82 suggested dementia.

Seizure types were classified as focal seizures, focal to bilateral generalized tonic-clonic seizures, and tonic-clonic seizures of unknown onset or unclassified based on semiology and interictal electroencephalogram findings.^[25]^

Sample size estimates: In projecting statistical power, as there are no competing epilepsy studies in this setting and the MakCHS experience with the epilepsy. For the quantitative study, we will enroll a sample of size 400, we will have power of at least 0.80 for a 2-sided test of interaction effect with Type I error of 0.05. This assumes a prevalence of 50% due to lack of prior studies and adjusted for 10% non-response to the study.

### 2.6. Data analysis

We used frequencies and proportions expressed as percentages to summarize categorical variables and median to summarize continuous variables. Except age, all the other variables were categorical. The prevalence of seizure in the past 30 days was estimated with 95% confidence interval (CI) and presented overall and by all categorical variables. Also, we analyzed the cognitive levels of the study participants by estimating the proportions with normal, impaired, and abnormal (dementia) cognitive function. The proportions with the 3 different cognitive function levels were presented overall and by demographic characteristics and compared between different categories using Fisher’s exact test. Due to small numbers of participants with dementia (9 out of 405 assessed for cognitive function), these were combined with those who were abnormal, and we referred to this category as cognitive deficit. The prevalence with cognitive deficit was estimated with 95% CI and presented overall and by demographic characteristics. To determine factors associated with seizure prevalence and cognitive deficit, a generalized linear model (GLM) with family binomial and a log-link function was used. Using the GLM, we estimated prevalence ratios (PR) with 95% CIs and used these to quantify the magnitude of the associations. Both bi-variate and multi-variate analyses were performed using the GLM. We subjected all factors with bi-variate *P* values of <.2 to a multiple GLM (multivariate analysis) and built the model using a backward elimination method while checking for multicollinearity. Results from multivariate analysis are presented as adjusted estimates. All the analyses were performed using STATA version 14.

### 2.7. Ethical considerations

The study protocol was approved by the Makerere University School of Medicine Research and Ethics Committee (SOMREC), approval number: 2020-172 and the Uganda National Council for Science and Technology, approval number: HS970ES. All patients or their caregivers provided written informed consent to participate in the study.

## 3. Results

### 3.1. Socio-demographic characteristics of the study participants

**Figure 1.**
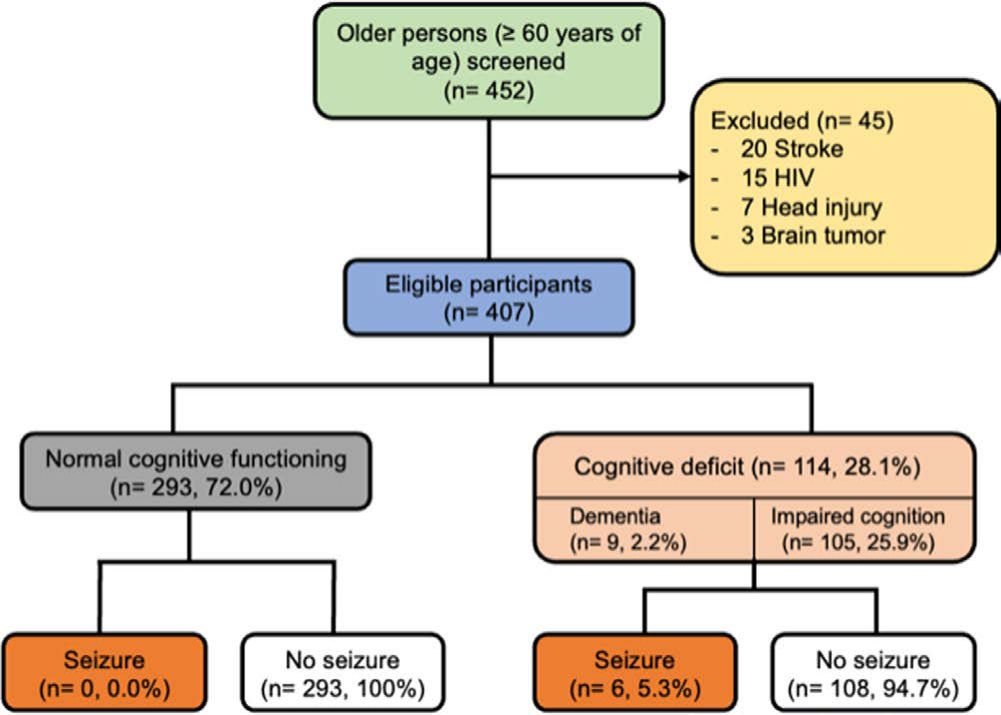
Showing the study participant enrollment.

Of the 452 participants screened, a total of 407 participants, with a median (inter-quartile range) age of 67 (64–73) years met eligibility criteria and were enrolled into the study (Fig. 1). Of these, the majority were female (n = 292, 71.7%), single (n = 186, 46.3%), unemployed (n = 249, 61.3%), had received primary education (n = 216, 53.3%), and were from districts around the study site, that is, Kampala (n = 164, 40.3%), Wakiso (n = 156, 38.3%), and Mukono (n = 19, 4.7%), see Table 1.

**Table 1:** Distribution of socio-demographic characteristics of study participants.

Characteristics	Frequency	Percentage
**Sex**	**N = 407**	
Male	115	28.3
Female	292	71.7
**Age, yrs**	**N = 404**	
60-64	121	30.0
65-69	123	30.5
70-74	89	22.0
75+	71	17.6
Median (IQR), yrs	67 (64, 73)	
**Marital status**	**N = 402**	
Divorced	62	15.4
Married	154	38.3
Single	186	46.3
**Employment status**	**N = 406**	
Employed	157	38.7
Unemployed	249	61.3
**Education status attained**	**N = 405**	
Never attended school	27	6.7
Primary school	216	53.3
Secondary school	106	26.2
University level	56	13.8
**District**	**N = 407**	
Kampala	164	40.3
Wakiso	156	38.3
Mukono	19	4.7
Luweero	6	1.5
Mpigi	8	2.0
Buikwe	5	1.2
Butambala	5	1.2
Nakasongola	5	1.2
Mityana	5	1.2
Other	34	8.4

IQR = inter-quartile range.

### 3.2. Prevalence of seizures

**Figure 2.**
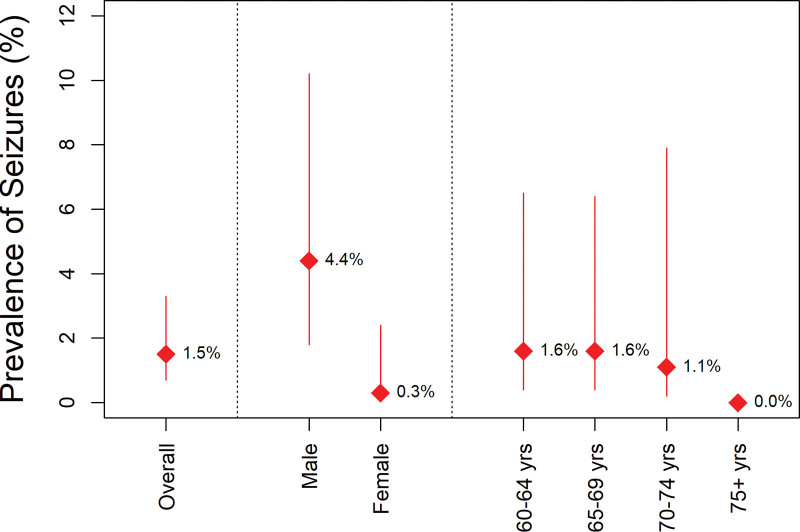
Prevalence of seizure in the past 30 days, overall and by sex and age group.

Overall, the prevalence of seizure among the study participants was 1.5% (95% CI: 0.7–3.3). All those who experienced seizures were unemployed. The prevalence of seizure was significantly higher among males compared to female participants (4.3% vs 0.3%, *P* = .02), Seizure prevalence did not significantly differ across age groups over the past 30 days by age and sex; see Figure 2. The prevalence of seizures was highest in Mukono district at 10.5% (95% CI: 2.8–39.0), Table 2.

**Table 2:** Prevalence of seizure and associated factors.

Characteristics	Number with seizuren/N	Prevalence of seizure(95% CI)%	Prevalence ratio(95% CI)	*P* value
**Overall**	6/407	1.5 (0.7, 3.3)		
**Sex:**				
Male	5/115	4.3 (1.8, 10.2)	Ref.	
Female	1/292	0.3 (0.05, 2.4)	0.08 (0.01, 0.67)	**.020**
**Age (yrs**)				
60-64	2/121	1.7 (0.4, 6.5)	Ref.	
65-69	2/123	1.6 (0.4, 6.4)	0.98 (0.14, 6.87)	.987
70-74	1/89	1.1 (0.2, 7.9)	0.68 (0.06, 7.38)	.751
≥75	0/71	…	…	…
**Marital status**				
Divorced	1/62	1.6 (0.2, 11.3)	Ref.	
Married	4/154	2.6 (1.0, 6.8)	1.61 (0.18, 14.12)	.667
Single	1/186	5.4 (0.1, 3.8)	0.33 (0.02, 5.25)	.435
**Education status attained**				
Never attended School	0/27	…		
Primary school	2/216	0.9 (0.2, 3.7)	Ref.	
Secondary school	2/106	1.9 (0.5, 7.4)	2.04 (0.29, 14.27)	.473
University level	2/56	3.6 (0.9, 13.9)	3.86 (0.56, 26.78)	.172
**District**				
Kampala	1/164	0.6 (0.1, 4.3)	Ref.	
Wakiso	2/156	1.2 (0.3, 5.1)	2.10 (0.19, 22.96)	.542
Mukono	2/19	10.5 (2.8, 39.0)	17.26 (1.64, 181.55)	**.018**
Other	1/64	1.5 (0.2, 10.3)	2.41 (0.15, 38.0)	.531
**Cognitive function**				
Normal	0/291	0		
Abnormal	6/114	5.3 (2.4, 11.5)	…	…

CI = confidence interval.

### 3.3. Factors associated with seizure among the study participants

The prevalence of seizures was 92% lower among female participants compared to their male counterparts (PR: 0.08, 95%CI: 0. 01–0.67, *P* = .020). The prevalence of seizures was nearly 17-fold higher in participants from Mukono districts compared to participants from Kampala city (PR: 17.26, 95%CI: 1.64–181.55, *P* = .018), Table 2. Other covariates were not statistically significantly associated with seizure prevalence. The prevalence of seizure was 5.3% among those with abnormal cognitive function and 0% among those with normal function.

### 3.4. Seizures semiology

All 6 participants with seizure reported generalized tonic-clonic seizure type.

### 3.5. Cognitive function

**Figure 3.**
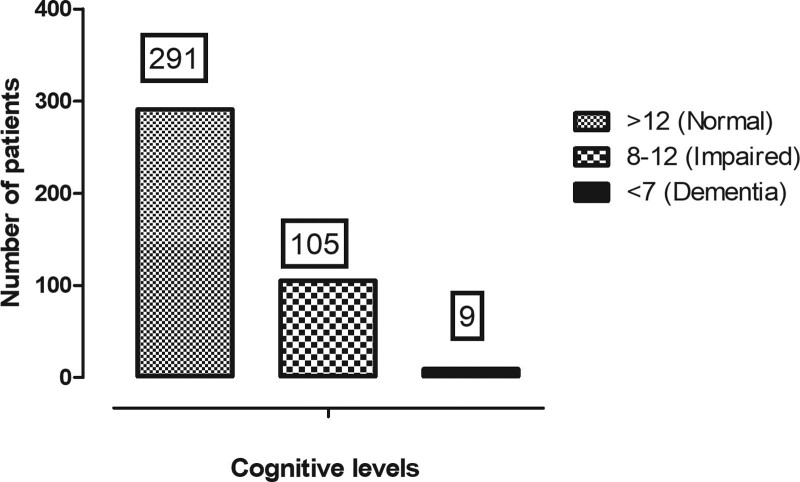
Distribution of cognitive levels.

Of the 407 participants, 405 were evaluated for cognitive levels. Of these, 114 (28.1%, 95% CI: 24.1–32.9) had a cognitive deficit; 9 (2.2%) dementia, and 105 (25.9%) impaired cognition, Figure 3.

### 3.6. Association between cognitive function and sociodemographic characteristics

At univariable analysis (Table 3), there was trend towards a higher prevalence of impaired cognition and dementia among female participants compared to their male counterparts (*P* = .053). The prevalence of dementia and cognitive impairment were significantly higher in participants aged 75 years or older (37.5% dementia vs 34.6% impaired cognition vs 10.7% normal cognition, *P* < .001). Also, a higher proportion of participants with dementia (88.9%) and impaired cognition (77.1%) were unemployed. The observed difference was statistically significant (*P* < .001). A significantly higher proportion of participants with dementia had attended post-primary education (66.7%) compared to those with impaired cognition (33.6%) and those with normal cognition (41.6%).

**Table 3:** Distribution of cognitive levels by demographic characteristics.

	Cognitive function			
Characteristics	Normal(Score > 12)n (%)	Impaired(Score: 8 —12)n (%)	Dementia(Score ≤ 7)n (%)	*P* value
**Overall**	291 (71.9%)	105 (25.9%)	9 (2.2%)	
**Sex:**	**N = 291**	**N = 105**	**N = 9**	
Male	73 (25.1)	39 (37.1)	3 (33.3)	**.053**
Female	218 (74.9)	66 (62.9)	6 (66.7)	
**Age at onset of seizures (yrs**)	**N = 290**	**N = 104**	**N = 8**	
60-64	102 (35.2)	15 (15.4)	2 (25.0)	**<.001**
65-69	95 (32.8)	26 (25.0)	2 (25.0)	
70-74	62 (21.4)	26 (25.0)	1 (12.5)	
≥75	31 (10.7)	36 (34.6)	3 (37.5)	
**Marital status**	**N = 289**	**N = 103**	**N = 9**	
Divorced	45 (15.6)	16 (15.5)	1 (11.1)	.979
Married	113 (39.1)	38 (36.9)	3 (33.3)	
Single	131 (45.3)	49 (47.6)	5 (55.6)	
**Employment status**	**N = 291**	**N = 105**	**N = 9**	
Unemployed	159 (54.6)	81 (77.1)	8 (88.9)	**<.001**
Employed	132 (45.4)	24 (22.9)	1 (11.1)	
**Education status attained**	**N = 291**	**N = 104**	**N = 9**	
Never attended school	13 (4.5)	14 (13.5)	0 (0.0)	.024
Primary school	157 (54.0)	55 (52.9)	3 (33.3)	
Secondary school	76 (26.1)	25 (24.0)	5 (55.6)	
University level	45 (15.5)	10 (9.6)	1 (11.1)	

At multivariable analysis (Table 4), cognitive deficit was independently associated with female gender (adjusted prevalence ratio [aPR]: 0.61, 95%CI: 0.44–0.85, *P* = .003), formal employment (aPR: 0.53, 95%CI: 0.35–0.81, *P* = .003), age between 70 and 74 years (aPR: 1.69, 95%CI: 1.00–2.86, *P* = .049), and 75 years or older (aPR: 2.81, 95%CI: 1.71–4.61, *P* = .001).

**Table 4:** Factors associated with cognitive deficit among the participants.

Characteristics	Number with cognitive deficit, n/N	Prevalence of cognitive deficit, (95% CI), %	Crude PR(95% CI)	*P* value	Adjusted PR(95% CI)	*P* value
**Overall**	114/405	28.1 (24.1, 32.9)				
**Sex:**						
Male	42/115	36.5 (28.7, 46.5)	Ref.		Ref.	
Female	72/292	24.8 (20.3, 30.3)	0.68 (0.50, 0.93)	0.016	0.61 (0.44, 0.85)	**.003**
**Age at onset of seizures (yrs**)						
60-64	18120	15.0 (9.8, 23.0)	Ref.		Ref.	
65-69	28/123	22.8 (16.4, 31.5)	1.52 (0.89, 2.59)	0.127	1.36 (0.80, 2.31)	.251
70-74	27/89	30.3 (22.1, 41.6)	2.02 (1.19, 3.43)	0.009	1.69 (1.00, 2.86)	.049
75+	39/71	55.7 (45.2, 68.7)	3.71 (2.31, 5.97)	0.001	2.81 (1.71, 4.61)	.001
**Employment status**						
Unemployed	89/248	35.9 (30.4, 42.4)	Ref.		Ref.	
Employed	25/157	15.9 (11.1, 22.8)	0.44 (0.30, 0.66)	0.001	0.53 (0.35, 0.81)	**.003**
**Education status attained**						
None	14/27	51.9 (36.1, 74.6)	Ref.		Ref.	
Primary	58/215	27.0 (21.7, 33.6)	1.92 (1.26, 2.94)	0.003	1.44 (0.94, 2.20)	.097
Secondary	30/106	28.3 (20.9, 38.3)	1.05 (0.72, 1.53)	0.802	1.14 (0.78, 1.66)	.514
University level	11/56	19.6 (11.6, 33.4)	0.73 (0.41, 1.29)	0.278	0.68 (0.41, 1.13)	.132
**Marital status**						
Divorced	17/62	27.4 (18.3, 41.1)	Ref.			
Married	41/154	26.6 (20.5, 34.6)	0.97 (0.60, 1.57)	0.905		
Single	54/185	29.2 (23.3, 36.5)	1.06 (0.67, 1.69)	0.791		
**District**						
Kampala	41/163	25.2 (19.3, 32.8)	Ref.			
Wakiso	44/156	28.4 (22.1, 36.5)	1.13 (0.78, 1.62)	0.515		
Mukono	7/19	36.8 (20.4, 66.4)	1.46 (0.77, 2.79)	0.247		
Others	22/68	32.4 (22.9, 45.6)	1.29 (0.83, 1.98)	0.256		

CI = confidence interval.

## 4. Discussion

This study investigated cognitive functioning and the prevalence and factors associated with seizure and cognitive deficits among older persons attending an outpatient medical clinic in Uganda. Primary data on the prevalence of seizure disorders and cognitive deficits among older persons in sub-Saharan Africa still remains scarce.^[3,20]^ In this study, we report 2 main findings. First, we found a prevalence of seizure of about 1.5%, will all participants self-reporting have generalized tonic-clonic seizures. This finding is consistent with our recent systematic review which showed a pooled seizure prevalence of about 0.8% among a population of over 10,000 older persons in 4 sub-Saharan African countries.^[3]^ In this systematic review, seizure prevalence ranged from 0.02% to 3.2% across individual studies. Two studies conducted in Tanzania reported seizure prevalence of 0.02% in a community setting^[26]^ and 3.2% in the hospital setting.^[27]^ Two other studies from west African countries, both hospital-based cross-sectional studies reported seizure prevalence of 1.4% in Senegal^[28]^ and 1.35% in Niger.^[29]^ These few studies highlight the relatively higher prevalence of seizure in hospital-based, compared to community-based studies. This is consistent with our finding which showed a comparable prevalence. In hospital settings, patients are likely to be attending health-facilities for various reasons such as strokes, diabetes mellitus and various other co-morbidities which may increase the risk for seizures in this population.

Secondly, we found cognitive deficits in about 28.1% of the participants, with an overall prevalence of dementia at 2.2%. The prevalence of dementia is consistent with a previous estimate of dementia prevalence of about 1-3% among older persons in sub-Saharan Africa.^[20]^ In Uganda, a few studies have reported on the prevalence of cognitive deficits in diverse populations. Among older persons in western Uganda, severe cognitive impairment was observed in 28% of the participants.^[30]^ Another study from rural Uganda reported a dementia prevalence of 20% among the general population of persons older than 60 years.^[31]^ On the other hand, Mukisa and colleagues reported a 20% prevalence of dementia among patients who recently recovered from stroke.^[21]^

Several factors contribute to cognitive deficits in older persons. In the present study, female gender and formal employment were protective, meanwhile, advancing age significantly predicted higher prevalence risk of seizure. These observations are consistent with previous reports from Uganda and beyond.^[20,21,30,31]^ In this study, all cases of seizures were reported in patients with cognitive deficits.

Seizures may relate to disease severity and/or duration of dementia illness. In some studies, seizures noted after the onset of dementia were more likely to occur in later stages of the disease.^[32]^ However, other population studies found that neither disease duration nor age of onset were significant risks for seizures in AD patients.^[33–35]^ One possible explanation is that dementia severity, rather than disease duration, may be associated with risk of seizures. In prospective studies of patients with probable AD of mild severity, seizures occurred in 1.5% to 16% of patients over 1 to 8.5 years of follow-up.^[32,34]^ Dementia severity or worse performance on tests of orientation and information have been reported to be associated with an increased risk of seizures in AD patients.^[36]^

This study has some limitations. First, the sample size is small to generalize the results to the elderly population in Uganda. Secondly, we relied on self-reported description of the seizures by next of kin, which may not be accurate and may be influenced by recall bias. Thirdly, we did not collect data on other underlying medical co-morbidities such as diabetes mellitus, rheumatic diseases etc which may have confounded on the cognitive functioning and seizure prevalence. Fourthly, we did not explore the age at onset of seizure and some cases might have had seizure onset before the age of 60 years. However, this is a baseline study which provides insights into the burden of seizures among older persons and guides future studies.

## 5. Conclusions

In summary, among older persons attending an outpatient clinic in Uganda, we found about 28% participants to have cognitive deficits with up to about 2.2% having dementia. The overall prevalence of seizure was 1.5% and was 3.5-fold higher in participants with cognitive deficits compared to those with normal cognitive functioning. The high prevalence of cognitive deficits is of public health significance and merits further studies to inform routine screening for this disorders among in medical outpatient departments.

## Author contributions

Mark Kaddumukasa and EK conceptualized the study. Mark Kaddumukasa, FB, Micheal Kiyingi, LM, EK and MS designed the study. Mark Kaddumukasa, Micheal Kiyingi and FB curated the data. Mark Kaddumukasa and LM analyzed the data. Mark Kaddumukasa and FB drafted the manuscript. EK and MS critically reviewed the manuscript for intellectual content and supervised the study. All authors read and approved the final manuscript.

**Conceptualization:** Mark Kaddumukasa, Felix Bongomin, Micheal Kiyingi, Elly Katabira, Martha Sajatovic.

**Data curation:** Mark Kaddumukasa, Felix Bongomin, Levicatus Mugenyi, Micheal Kiyingi, Elly Katabira, Martha Sajatovic.

**Formal analysis:** Mark Kaddumukasa, Felix Bongomin, Levicatus Mugenyi.

**Funding acquisition:** Mark Kaddumukasa, Elly Katabira, Martha Sajatovic.

**Investigation:** Micheal Kiyingi.

**Methodology:** Mark Kaddumukasa, Felix Bongomin, Levicatus Mugenyi, Micheal Kiyingi, Martha Sajatovic.

**Project administration:** Mark Kaddumukasa.

**Supervision:** Martha Sajatovic.

**Writing – original draft:** Mark Kaddumukasa, Felix Bongomin, Levicatus Mugenyi, Micheal Kiyingi.

**Writing – review & editing:** Mark Kaddumukasa, Felix Bongomin, Levicatus Mugenyi, Elly Katabira, Martha Sajatovic.
